# The de novo assembly of a European wild boar genome revealed unique patterns of chromosomal structural variations and segmental duplications

**DOI:** 10.1111/age.13181

**Published:** 2022-03-02

**Authors:** Jianhai Chen, Jie Zhong, Xuefei He, Xiaoyu Li, Pan Ni, Toni Safner, Nikica Šprem, Jianlin Han

**Affiliations:** ^1^ Institutes for Systems Genetics Frontiers Science Center for Disease‐related Molecular Network West China Hospital Sichuan University Chengdu China; ^2^ Animal Husbandry and Veterinary Institute of Keqiao District Shaoxing Zhejiang China; ^3^ Faculty of Agriculture University of Zagreb Zagreb Croatia; ^4^ Centre of Excellence for Biodiversity and Molecular Plant Breeding (CoE CroP‐BioDiv) Zagreb Croatia; ^5^ International Livestock Research Institute Nairobi Kenya; ^6^ CAAS‐ILRI Joint Laboratory on Livestock and Forage Genetic Resources Institute of Animal Science Chinese Academy of Agricultural Sciences Beijing China

**Keywords:** meiotic sex chromosome inactivation, reference genome, *Sus scrofa*, whole genome sequencing

## Abstract

The rapid progress of sequencing technology has greatly facilitated the de novo genome assembly of pig breeds. However, the assembly of the wild boar genome is still lacking, hampering our understanding of chromosomal and genomic evolution during domestication from wild boars into domestic pigs. Here, we sequenced and de novo assembled a European wild boar genome (ASM2165605v1) using the long‐range information provided by 10× Linked‐Reads sequencing. We achieved a high‐quality assembly with contig N50 of 26.09 Mb. Additionally, 1.64% of the contigs (222) with lengths from 107.65 kb to 75.36 Mb covered 90.3% of the total genome size of ASM2165605v1 (~2.5 Gb). Mapping analysis revealed that the contigs can fill 24.73% (93/376) of the gaps present in the orthologous regions of the updated pig reference genome (Sscrofa11.1). We further improved the contigs into chromosome level with a reference‐assistant scaffolding method. Using the ‘assembly‐to‐assembly’ approach, we identified intra‐chromosomal large structural variations (SVs, length >1 kb) between ASM2165605v1 and Sscrofa11.1 assemblies. Interestingly, we found that the number of SV events on the X chromosome deviated significantly from the linear models fitting autosomes (*R* ^2^ > 0.64, *p* < 0.001). Specifically, deletions and insertions were deficient on the X chromosome by 66.14 and 58.41% respectively, whereas duplications and inversions were excessive on the X chromosome by 71.96 and 107.61% respectively. We further used the large segmental duplications (SDs, >1 kb) events as a proxy to understand the large‐scale inter‐chromosomal evolution, by resolving parental‐derived relationships for SD pairs. We revealed a significant excess of SD movements from the X chromosome to autosomes (*p* < 0.001), consistent with the expectation of meiotic sex chromosome inactivation. Enrichment analyses indicated that the genes within derived SD copies on autosomes were significantly related to biological processes involving nervous system, lipid biosynthesis and sperm motility (*p* < 0.01). Together, our analyses of the de novo assembly of ASM2165605v1 provides insight into the SVs between European wild boar and domestic pig, in addition to the ongoing process of meiotic sex chromosome inactivation in driving inter‐chromosomal interaction between the sex chromosome and autosomes.

## INTRODUCTION

Pigs (*Sus scrofa domesticus*) were domesticated from their wild boar ancestors ~10,000 years ago during the early Neolithic agricultural revolution in the Near East and Central China independently (Rothschild & Ruvinsky, 2011). As one of the most important sources of animal protein, pork production has long been valued in East and Southeast Asia and some European countries. The pig industry is also expected to increase steadily at both global and regional scales in the next few years (https://www.alliedmarketresearch.com/pork‐meat‐market, accessed 20 December 2021). In addition to their valuable meat, pigs can also provide leather, bristles and lard products. The economically and agriculturallly important features of pigs are majorly attributed to the long‐lasting and ongoing efforts of breeding management and directional selection, especially since the Industrial Revolution (Bosse et al., 2014). Evidence based on comparative genomic analyses between wild boars and domestic pigs has shown the strong artificial selection that dramatically promoted the phenotypic transformation following domestication in both Europe and Asia (Li et al., 2010, 2017; Moon et al., 2015; Rubin et al., 2012; Wang et al., 2015; Yang et al., 2014).

In recent decades, pigs have also draw attention from medical field for translational research. Owing to their physiological and anatomical similarity to humans, pigs can serve as medical models for multiple human diseases. For example, there are reports on pig models for xenotransplantation (Blusch et al., 2002; Mariscal et al., 2018), wound healing (Sullivan et al., 2001), dental and orofacial research (Wang et al., 2007), gastrohelcoma (Tian et al., 2009), hearing loss (Guo & Yang, 2015) and neurodegenerative disorders (Holm et al., 2016). Research efforts in recent years have made tremendous strides toward the genome‐wide editing of pigs, including the inactivation of porcine endogenous retroviruses (Yang et al., 2015) and germline engineering (Yue et al., 2021). These various medical explorations using pigs as large animal models strongly indicate the high potential of pigs in helping to tackle human medical problems.

Apart from their economic and medical value, pigs and their wild counterparts can also serve as a great model for evolution and population genetic studies. Just as J.B.S. Haldane, one of founders of population genetics and neo‐Darwin synthesis in 1930s and 1940s, proposed, ‘One of the most hopeful fields for the study of evolution is the domestication of animals and perhaps also of plants’ (Lickliter & Ness, 1990; Haldane, 1954). Even the establishment of Darwin’s natural selection theory had been gleaned from extensive studies on the artificial selection of phenotypic variations in domesticated species (Darwin, 1875). In the current genomic era, domesticated species have been extensively sequenced, leading to the accumulation of abundant genomic data that are second to human population genomic data only. Unlike other popular domestic animals, whose wild counterparts are either limited in natural distribution (chickens and yak), endangered in population size (goats and sheep) or even extinct (horses and cattle), pigs are distinctive owing to their strong ability for widespread adaptation and long‐range migration, thereby leading to their population flourishing for both wild boars and domestic breeds (Chen et al., 2018a; Johann et al., 2020; Rothschild & Ruvinsky, 2011).

The high‐quality genome assemblies of domestic breeds/varieties have greatly promoted our understanding of numerous basic biological questions across a wide range of animals and plants, including the genetic bases of complex phenotypes in horses, chickens and pigs (Liu et al., 2022; Rubin et al., 2012; Wang et al., 2020), the ancient evolutionary processes of *S. scrofa* (Ai et al., 2015), genomic diversity in pigs (Li et al., 2017) and the origin and evolution of new genes in pigs and plants (Chen et al., 2019; Zhang et al., 2019b). Despite the fundamental role of genome assemblies in biological studies, high‐quality assemblies are limited to well‐known domestic pig breeds. For example, all currently available chromosome‐level assemblies were from domestic breeds, including Duroc (Groenen et al., 2012; Warr et al., 2020), Bama (Zhang et al., 2019a), Luchuan (Yang et al., 2019), Ningxiang (Ma et al., 2022) and Meishan (Zhou et al., 2021), whereas the scaffold‐level assemblies also came from domestic breeds, including Wuzhishan (Fang et al., 2012), Large White, Landrace, Berkshire, Hampshire, Pietrain, Bamei, Jinhua, Rongchang and Tibetan (Li et al., 2017). For wild boars, there are some short‐read Illumina sequences (Bosse et al., 2015; Frantz et al., 2015; Groenen, 2016), but no assembly of a genome based on long‐reads or long‐range sequencing. In this study, we de novo assembled a European wild boar genome using Linked‐Reads sequencing (Marks et al., 2019; Weisenfeld et al., 2017; Zheng et al., 2016). This assembly provides insight into evolutionary patterns between wild boars and domestic pigs on both inter‐ and intra‐chromosomal scales.

## MATERIALS AND METHODS

### DNA sampling, sequencing and assembly

Genomic DNA was extracted from the muscle tissue of a European male wild boar (France), which was collected during the regular hunting season according to national laws. The Linked‐Reads approach developed by 10× Genomics was used for sequencing the genomic DNA (Marks et al., 2019). Briefly, the Linked‐Reads method can provide long‐range information for genomic short reads by leveraging microfluidics to partition and barcode the high‐molecular‐weight DNA. Following the recommendations of the sequencing platform (10× Genomics), we obtained ~56× depths of sequencing reads. The mitogenome was constructed using GetOrganelle (Jin et al., 2020) and used as a query to search the NCBI nucleotide database to confirm whether the sample was a wild boar. The de novo assembly of whole genome data was performed using supernova v2.1.1 (Weisenfeld et al., 2017) downloaded from the official website of 10× Genomics, with default parameters. Finally, the contig‐level assembly of ASM2165605v1 was upgraded into chromosomal scale with ragtag (Alonge et al., 2019).

### Identification of structural variations

We identified structural variation (SVs), including deletions, insertions, duplications and inversions, using syri v1.4 (Goel et al., 2019). We further filtered out the short SVs of <1 kb and focused only on the continuous sequences for subsequent analyses. To understand whether the numbers of SVs were significant, we conducted regression analysis using the number of SVs against the length of relevant chromosomes. If the SVs are neutral and uniformly distributed, we may expect a linear pattern of the number of SVs conditional on chromosomal length. The regression analysis was performed with R packages. The biological processes of gene sets were analyzed using clusterprofiler (Wu et al., 2021).

### Continuity analysis and mapping rate

To understand whether our new ASM2165605v1 assembly can fill the remaining gaps in the pig reference genome, Sscrofa11.1, we used MashMap aligner to achieve the ‘one‐to‐one’ syntenic region identification at first (identity over 90%) (Jain et al., 2018). bedtools software (Quinlan & Hall, 2010) was further used to identify regions with gaps (represented by ‘N’) in Sscrofa11.1, but with uninterrupted sequences in ASM2165605v1. To understand whether our ASM2165605v1 can fill more gaps than previous assemblies of major European pig breeds, we repeated the above pipeline and compared the gap‐filling rates among these assemblies. The assemblies of Asian pig breeds were not used to avoid potential misalignments. A comparison of mapping rates between different assemblies of European pig breeds and ASM2165605v1 was conducted using BWA‐MEM (Li & Durbin, 2009). The marking of duplicate alignments was done using the samtools suite (Li et al., 2009).

### Genomic annotation for genes and repeats

We annotated ASM2165605v1 for its gene and repeat contents. The coding and non‐coding genes were annotated by following the methods of previous assemblies (Groenen et al., 2012; Warr et al., 2020). Briefly, the protein‐coding genes were annotated using the MAKER2 pipeline (Cantarel et al., 2008) by jointly using three methods, comprising RNAseq mapping, de novo predictions and homologous gene searching. The pair‐ended Illumina RNA‐seq data of wild boars were downloaded from the BioProject of PRJEB3197 at NCBI. The de novo read mapping and assembly were conducted to obtain transcripts with packages of star (Dobin et al., 2013) and trinity (Haas et al., 2013). The genome‐wide repeats were identified using repeatmasker with RM and Repbase repeats.

### Identification of segmental duplications

We designed a pipeline, as visualized in Figure 4, to perform the identification of segmental duplications (SDs). Briefly, the whole genome alignment was performed by comparing Sscrofa11.1 against ASM2165605v1 using the LASTAL alignment tool (Hamada et al., 2017). The ‘many‐to‐one’ alignments over 1000 bp were kept as the domestic SD pair. To understand which one was the parental copy within a SD pair, we categorized the SDs into two types, the boundary‐derived SD (bSD) and the internal‐derived SD (iSD). For the iSD, it was easy to identify the parent‐derived relationship, considering the feasible assumption that the synteny length of a parental copy should be longer than that of a derived copy. For the bSD, we determined the copying direction using BLASTN mapping of the SD pair against the orthologous copy in ASM2165605v1. The copy with a higher nucleotide identity of BLASTN comparison (>90%) was determined to be the parental copy because the distance between orthologous copies should be shorter than that between the derived copy and homologous copy. After determining the copying direction of SDs, we used the linear regression to fit the number of SDs at the inter‐chromosomal level. The clusterprofiler tool was used to conduct the over‐representation test and gene‐set enrichment analysis of Gene Ontology (Wu et al., 2021).

## RESULTS

### The sequencing and de novo assembling

The wild boar in this study was confirmed to be genetically nearest to European wild boar (FJ237002.1) based on complete mitogenomes with only two mismatches and a DNA identity of 99.9% (Figure S1). All other populations, including European local and commercial pigs as well as Asian wild boars and domestic pigs, showed a relatively low identity with the mitogenome assembled in this study.

For genomic data, in total, we obtained 1,696,695,959 linked reads, with 92.23% of them showing MapQ ≥30. We generated the de novo assembly of the European wild boar, entitled ASM2165605v1, using supernova (Weisenfeld et al., 2017), and kept the 13,542 contigs longer than 1000 bp. Among these contigs, there were 289 contigs longer than 100 kb, 77 contigs longer than 10 Mb and eight contigs longer than 50 Mb. The contig N50 value was 26.09 Mb, suggesting a high‐level of continuity empowered by long‐range information of the linked reads. Considering the close relationship between European wild boars and domestic pig breeds such as Large White, Berkshire, Landrace, Pietrain, Duroc and Hampshire (Frantz et al., 2015), we further examined whether ASM2165605v1 and contig‐level assemblies of other European pig breeds can fill the gaps remaining in the current pig reference genome (Sscrofa11.1; Warr et al., 2020).

**FIGURE 1:**
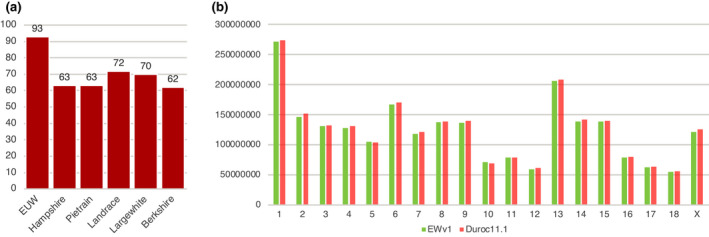
(a) The number of gaps which are filled by the assemblies of ASM2165605v1 and other European pig breeds. (b) The comparison of non‐missing lengths for all chromosomes of ASM2165605v1 and Sscrofa11.1 assemblies

Rigorous ‘one‐to‐one’ orthologous mapping was conducted using MashMap (Jain et al., 2018) by focusing on orthologous segments with over 90% identity between the assemblies of European breeds (Li et al., 2017), ASM2165605v1 and Sscrofa11.1. We revealed that the ASM2165605v1 contigs can fill more gaps in Sscrofa11.1 (93/376) than the current five assemblies of other European breeds (Figure 1a), suggesting that the continuity of the ASM2165605v1 assembly was better than those of all of the assemblies of other European breeds. Because extensive reports have established the close evolutionary relationship between local populations of wild boars and domestic breeds from Europe (Chen et al., 2018b; Frantz et al., 2013, 2015), the contigs of ASM2165605v1 could be ordered with the assistance of Sscrofa11.1 under the assumption of there being no large‐scale inversion between the contigs. We anchored the contigs of ASM2165605v1 with the ‘scaffold’ function of ragtag (Alonge et al., 2019) and achieved a scaffold N50 of 4.24 kb after filtering out unplaced contigs. We further estimated sequence lengths for chromosomes with gapless DNA in ASM2165605v1 and compared them with the gapless lengths of Sscrofa11.1 using *l*_r_ (*l*_r_ = ASM2165605v1_chr_length_/Sscrofa11.1_chr_length_). We found *l*_r_ ratios ranging from 0.962 to 1.023 (Figure 1b), suggesting highly comparable genome coverages between ASM2165605v1 and Sscrofa11.1. Interestingly, chromosomes 5 and 10 were longer in ASM2165605v1 than in Sscrofa11.1, although all the remaining chromosomes demonstrated longer coverages in Sscrofa11.1 than in ASM2165605v1.

We also compared the mapping rates of six assemblies of European breeds (Sscrofa11.1, Hampshire, Pietrain, Landrance, Large White and Berkshire) and ASM2165605v1. We randomly chose the publicly available short‐read DNA re‐sequencing data of 10 European wild boars (Table S1) and mapped the cleaned reads to the six assemblies independently. We found that the mapping rates for all 10 wild boars were highest when using Sscrofa11.1 as a reference (median 97.64%), supporting the high quality of the most updated pig reference genome (Warr et al., 2020). In addition, ASM2165605v1 had a higher median mapping rate (97.45%) than the other five assemblies of domestic breeds except Sscrofa11.1, suggesting its potential contribution to improving the overall continuity of pig pan‐genomes.

To annotate the genome‐wide protein‐coding genes, we jointly applied three commonly used methods, including transcriptome alignment, de novo gene prediction and sequence homology‐based predictions. In total, we obtained 21,400 protein‐coding genes, which accounted for 1.3% of ASM2165605v1 (Table 1). We also annotated non‐coding RNAs and genomic repeats (Table 2). In total, 0.273, 1.36, 0.131 and 0.77% of ASM2165605v1 was annotated as miRNA, tRNA, rRNA and snRNA respectively. Over 44% of ASM2165605v1 was identified as containing DNA repeats, including LINE, SINE, LTR, Satellite and unknown types of repeats, similar to previous reports on pig reference genome (Groenen et al., 2012; Warr et al., 2020).

**TABLE 1:** The annotated protein‐coding genes and non‐coding genes in ASM2165605v1

Type	Subtype	Count	Average length (bp)	Total length (bp)	Percentage of genome
Coding genes		21,400	34,328	32,493,688	1.3014
miRNA		861	79	68,172	0.2730
tRNA		4471	76	338,344	1.3551
rRNA	rRNA	135	242	32,614	0.1306
	18S	9	1506	13,550	0.543
	28S	3	1610	4829	0.0193
	5.8S	6	154	925	0.0037
	5S	117	114	13,310	0.0533
snRNA	snRNA	1697	113	192,366	0.7705
	CD‐box	294	92	26,999	0.1081
	HACA‐box	277	135	37,347	0.1496
	Splicing	1100	112	123,581	0.004950
	scaRNA	26	171	4439	0.000178

**TABLE 2:** The annotated genomic repeats and their summaries in ASM2165605v1

Type	Repbase TEs		Other TEs		De novo		Combined TEs	
	Length (bp)	Percentage in genome	Length (bp)	Percentage in genome	Length (bp)	Percentage in genome	Length (bp)	Percentage in genome
DNA	74,618,563	2.99	3,922,533	0.16	28,749,938	1.15	76,142,807	3.05
LINE	486,903,714	19.5	227,213,753	9.1	551,923,837	22.11	665,121,705	26.64
SINE	22,342,313	0.89	0	0	25,909,379	1.04	35,962,746	1.44
LTR	132,835,795	5.32	6,568,546	0.26	2,616,79,411	10.48	318,961,338	12.77
Satellite	8,716,245	0.35	0	0	4,100,318	0.16	8,849,865	0.35
Unknown	1,147,190	0.05	9942	0	1,230,106	0.05	2,387,238	0.1
Total	726,563,820	29.1	237,714,774	9.52	873,592,989	34.99	1,107,425,699	44.35

Note

DNA refers to DNA transposons whereas LINE/SINE/LTR are retrotransposons. The TEs represents Transposable elements.

### The excess of inversions and duplications but the deficiency of deletions and insertions on the X chromosome

**FIGURE 2:**
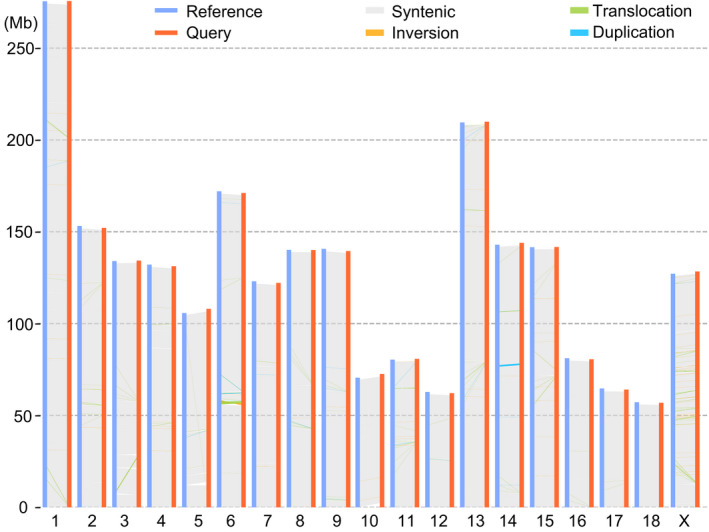
The structural variations between ASM2165605v1 and Sscrofa11.1 assemblies inferred with SyRI using default parameters. The four types of variations are shown in different colors

To understand the intra‐chromosome variations based on structural variations (SVs), we compared ASM2165605v1 with Sscrofa11.1 using SyRI, which is a synteny and rearrangement identifier (Goel et al., 2019) (Figure 2 and Table S2). After removing SVs shorter than 1 kb and focusing only on the continuous sequences, we identified a total of2700 SVs, including 1451 deletions, 833 insertions, 204 duplications and 212 inversions. Surprisingly, the longest inversion was found in chromosome 6 (1.49 Mb in Chr6:56947482‐58549530 of Sscrofa11.1), harboring 52 protein‐coding genes of six families inferred using the Markov Cluster Algorithm (van Dongen, 1991), of which only 12 had known functions (*ETFB*, *HAS1*, *LIM2*, *NKG7*, *PPP2R1A*, *SPACA6*, *VSIG10L*, *ZNF175*, *ZNF577*, *ZNF613*, *ZNF614* and *ZNF649*). Among these genes, *SPACA6* (sperm acrosome associated 6) was reported to be required for fusion of sperm with the egg membrane during fertilization (Noda et al., 2020).

**FIGURE 3:**
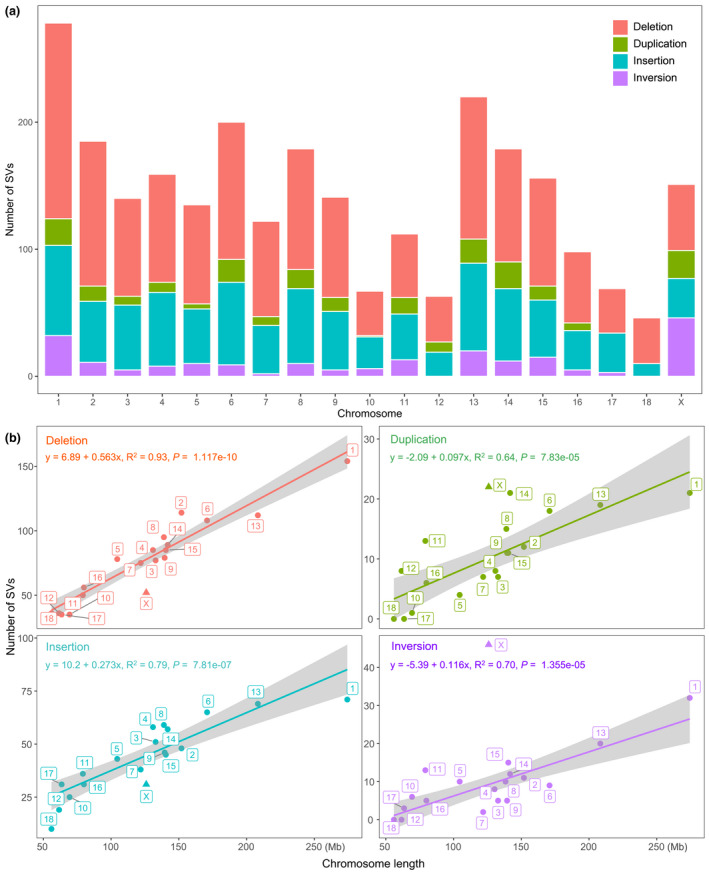
The numbers of structural variations across chromosomes (a) and the regression of the numbers of structural variations against the lengths of chromosomes (b)

The highest number of SVs was present in chromosome 1, the longest one in the pig genome (Figure 3a). To understand whether the numbers of SVs on different chromosomes followed a uniform distribution model with the null hypothesis that the longer the chromosome is, the higher number of the SVs, we further analyzed the number of SVs (>1 kb) in a rigid statistical framework against the lengths of chromosomes. Interestingly, compared with all autosomes, the X chromosome was significantly deficient in deletions and insertions but excessive in duplications and inversions (*p* < 0.001; Figure 3b). This opposite pattern suggested that the X chromosome may have a different level of sensitivity for SVs affecting chromosomal structural or functional conservation. If we consider the differences in effective population sizes (*Ne*) between chromosomes, our observation is even more striking. The *Ne* of X chromosome was roughly three‐quarters that of autosomes (Betrán et al., 2002), therefore the deviation of the X chromosome as an outlier would be even stronger. In detail, after adjusting the *Ne* estimates, the deficiency rates of deletions and insertions on the X chromosome were 66.14 and 58.41% respectively, whereas the excessive rates of duplications and inversions were relatively high, up to 71.96 and 107.61% respectively.

### The excessive traffic of segmental duplications ‘out of’ instead of ‘into’ the X chromosome

**FIGURE 4:**
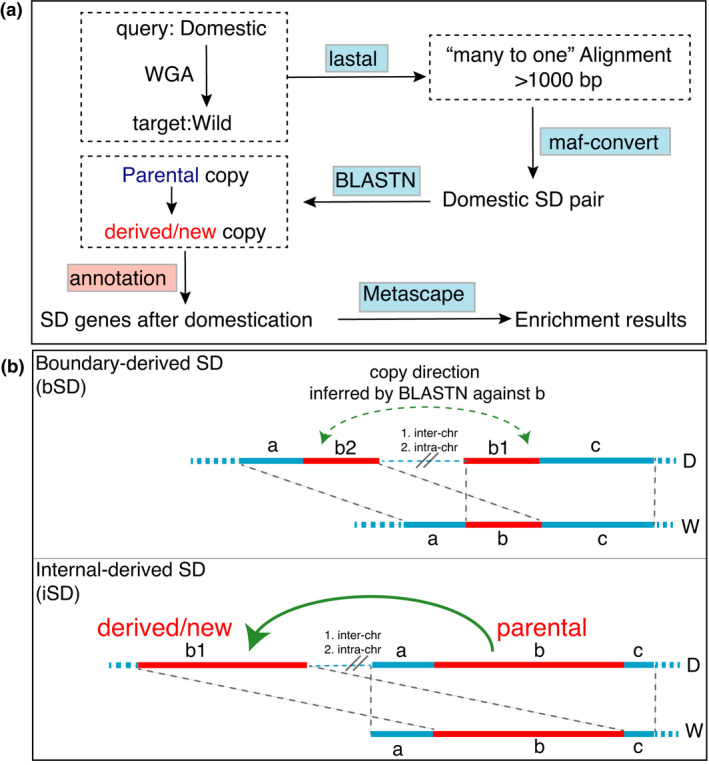
The pipeline designed for identifying the segmental duplications (SDs). (a) The flowchart of major software used and the overall processes. (b) The two types of SDs, which cover the boundary‐derived SD (bSD) and internal‐derived SD (iSD)

**FIGURE 5:**
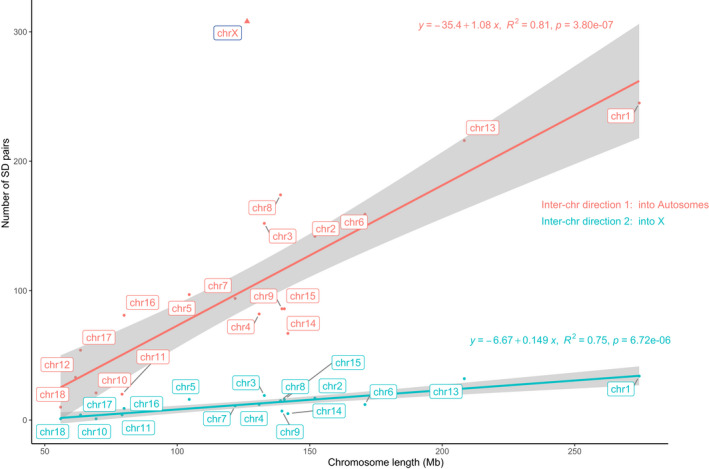
The regression of the numbers of SDs against the lengths of chromosomes. All numbers are inter‐chromosomal SD numbers. Red and blue show the directions ‘into autosomes’ and ‘into X chromosome’, respectively

As SVs only represent the intra‐chromosomal variations, whether there are inter‐chromosomal events involving large‐scale segmental duplications (SDs) is still unknown. Here, we developed an in‐house pipeline to identify the SDs between chromosomes (Figure 4). Based on the target chromosome, we defined two types of copying directions between chromosomes, including ‘into X’ and ‘into autosomes’ (Figure 5). Additionally, based on the source/parental chromosomes, these inter‐chromosomal types were further divided into three subtypes, which were ‘autosomes to X (A>X)’, ‘autosomes to autosomes (A>A)’, and ‘X to autosomes (X>A)’. We found the ‘A>X’ subtype to be significantly shaped by a linear model for all autosomes (*R* ^2^ = 0.75, *p* < 0.01, blue in Figure 5). Likewise, the ‘A>A’ subtype also demonstrated a linear model (*R* ^2^ = 0.81, *p* < 0.01, red in Figure 5). In contrast, the ‘X>A’ subtype, an excessive outlier of the linear model, was significantly different from both ‘A>A’ and ‘A>X’ subtypes. These patterns suggested that X chromosome had served as an excessive source to ‘export’ SDs into autosomes.

Gene duplications can be roughly classified into two types, RNA‐ and DNA‐mediated gene duplications, with the former arising through a mechanism termed retroposition or retroduplication (Kaessmann et al., 2009), whereas the latter is processed by several mechanisms, including unequal cross‐over and tandem, segmental, chromosomal and genome duplications (Kozlov, 2014). Previous reports have revealed the inter‐chromosomal events of retrogenes (RNA‐mediated gene duplications) in human, mouse, domestic pig and dog, and have found that X‐derived retrogenes in autosomes are excessive (Betrán et al., 2002; Chen et al., 2019; Gao et al., 2019). There are also findings on the excess of X‐derived genes on autosomes based on evidence of DNA‐mediated gene duplication (Vibranovski et al., 2009). The best‐known hypotheses to explain this underlying preference of the X‐derived movement involve sexual antagonism (Wu & Xu, 2003; Wyman et al., 2012) and meiotic sex chromosome inactivation (MSCI; Dai et al., 2006; Turner, 2007). Meiotic sex chromosome inactivation has been supported by a mouse model experiment, in which the evolutionarily new gene on an autosome can compensate for the function of parental gene in X chromosome owing to epigenetic silence during the male meiosis (Jiang et al., 2017). In this study, we provided clues that the excess of X‐derived SDs in autosomes could also be attributed to these molecular mechanisms, including MSCI.

**FIGURE 6:**
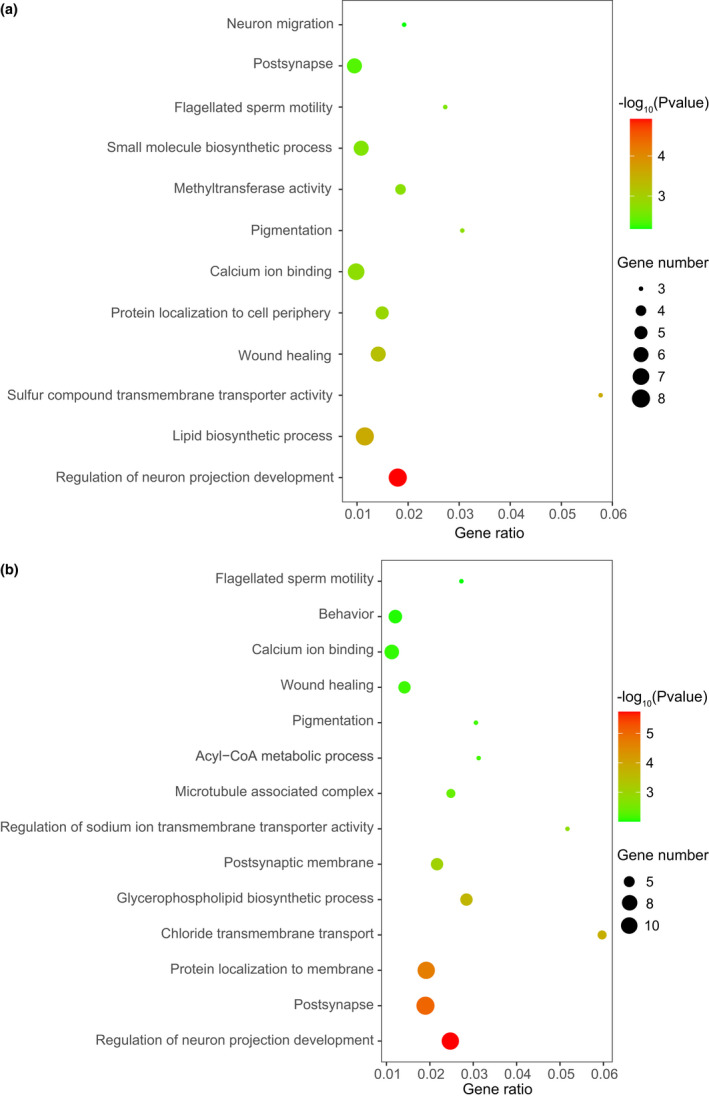
The enrichment analysis of biological processes using X‐derived autosomal genes (a) and all genes (b) related to SD movements. All processes are statistically significant (*p* < 0.01) as visualized with colors from green to red

If the hypothesis of MSCI driving the excess of X‐derived SDs is solid, we may expect that the genes covered by X‐derived SDs are involved in male meiosis‐related processes. Our enrichment analysis found that the genes linked with X‐derived SDs were significantly (*p* < 0.01) enriched in multiple processes involving the nervous system, metabolism and reproductive system (Figure 6). The enriched processes were stable for both X‐derived autosomal genes (Figure 6a) and all parental X‐genes and derived‐autosomal genes (Figure 6b). Specifically, the enriched biological process involving the reproductive system is flagellated sperm motility. This observation is probably relevant to MSCI, in which the epigenetic silence during mid‐ and post‐meiosis may impose an evolutionary force to drive the male‐meiotic advantageous genes to be transposed and expressed in autosomes.

## DISCUSSION

Exploring the advantage of the 10× Linked‐Reads sequencing, we de novo assembled the first, high‐quality genome, ASM2165605v1, of a European wild boar with contig N50 of 26.09 Mb. The contents of genes, repeats and non‐coding RNAs were highly similar between ASM2165605v1 and Sscrofa11.1. Notably, we recognized that, compared with the assemblies of several major European pig breeds stored in the Ensembl database, our ASM2165605v1 can fill the highest number of gaps in Sscrofa11.1. Overall, this novel ASM2165605v1 can therefore provide additional variations for the burgeoning pan‐genomes of wild boars and domestic pigs.

Comparative analyses between ASM2165605v1 and Sscrofa11.1 revealed an interesting pattern of SVs. Statistically, the deletions and insertions were deficient, whereas the duplications and inversions were excessive on the X chromosome. This finding is insightful for us to understand the intra‐chromosomal evolution at species level. Under the framework of the neutral evolution theory, we may expect the near linear distribution of SVs in chromosomes dependent on their lengths. Here, our observation of significant deficiency of the deletions and insertions in X chromosome suggests that this type of SV is under a stronger purifying selection than duplications and inversions. In contrast, as diversity is the genetic basis for positive selection, the excess duplications and inversions on the X chromosome advocate that they may have more chances to serve as a source of genetic variations for natural or artificial selection. Thus, our results support the selective heterogeneity of SVs on the X chromosome.

Meiotic sex chromosome inactivation is predicted to be an evolutionarily ancient mechanism critical for male reproductive processes. Owing to the importance of reproduction performance in domestic pigs, the domestication process provides a unique opportunity to test the impact of MSCI in this species. Here, we identified the frequent SDs by comparing ASM2165605v1 and Sscrofa11.1 assemblies and revealed a significant excess of SDs copied from the X chromosome to autosomes. Previous reports have proposed and validated the process of MSCI, which can drive the relocation of genes from the X chromosome to autosomes, to avoid the male meiotic silence of the X chromosome at both species level (Emerson et al., 2004; Jiang et al., 2017) and population level (Zhang & Tautz, 2021). Our observation is consistent with this well‐accepted theory.

In summary, we generated, for the first time, the de novo assembly of a European wild boar, to provide a basic genomic resource for future studies, and to improve, deepen and widen our understanding on genome evolution during domestication. Regardless of the questions on genomic diversity, population variations or even multiple evolutionary processes, the novel set of SVs and SDs identified from the comparison of two high‐quality wild boar and domestic pig assemblies may serve as an entry point for further exploration.

## CONFLICT OF INTEREST

The authors declare that they have no competing interests.

## Supporting information

Fig S1

Tables S1 and S2

## Data Availability

The sequence data and genome assembly of ASM2165605v1 can be accessed through NCBI GenBank BioProject code PRJNA791558 and assembly accession no. GCA_021656055.1 respectively.
