# Supplementary figures and images for: The de novo assembly of a European wild boar genome revealed unique patterns of chromosomal structural variations and segmental duplications
Source: Anim Genet. 2022 Mar 2;53(3):281–92. doi: 10.1111/age.13181 (PMC9314987; doi:10.1111/age.13181)

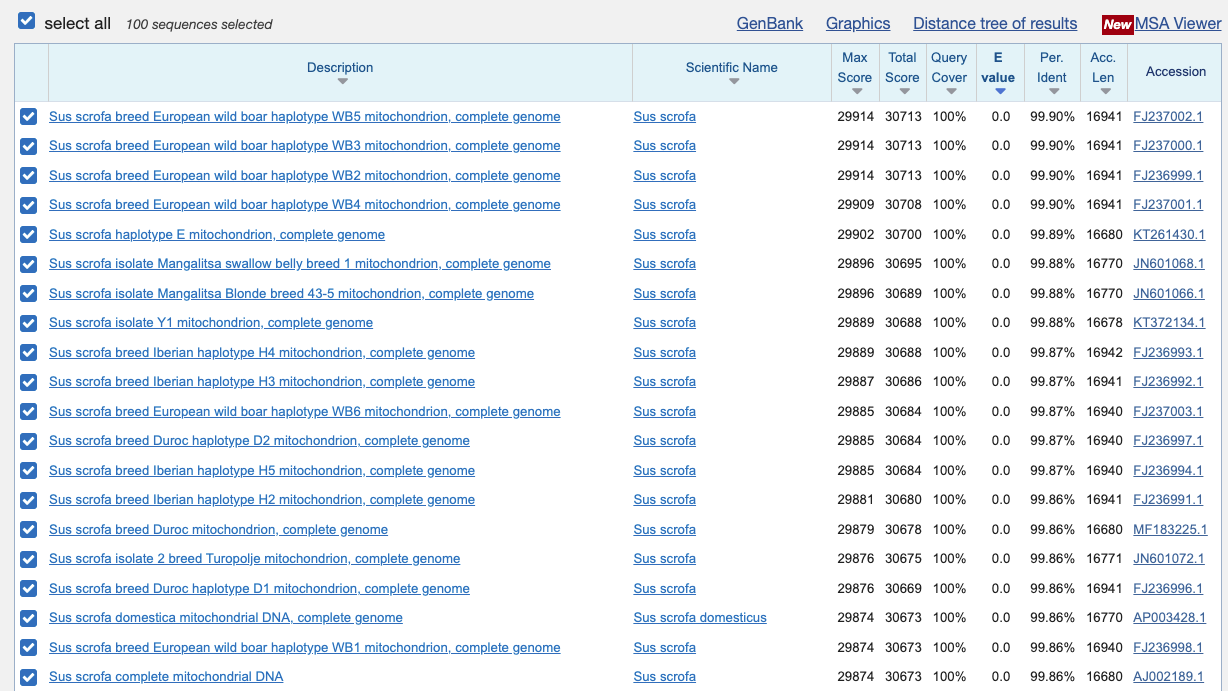

Supplement: Supplementary file 1 — Fig S1 [file AGE-53-281-s002.png]
